# Variations in DNA Repair Genes and Intratumoral Genetic Heterogeneity in Temozolomide‐Resistant Glioblastoma

**DOI:** 10.1155/humu/3430617

**Published:** 2026-04-10

**Authors:** Weixuan Chen, Cong Li, Yun Liu, Hengji Huang

**Affiliations:** ^1^ Department of Neurosurgery, Dongguan People′s Hospital, Dongguan, Guangdong Province, China, dgtpyy.com; ^2^ Department of Thoracic Surgery, Dongguan People′s Hospital, Dongguan, Guangdong Province, China, dgtpyy.com

**Keywords:** DNA repair genes, genetic heterogeneity, glioblastoma, personalized treatment, temozolomide resistance

## Abstract

**Background:**

Glioblastoma (GBM) is the most prevalent primary brain tumor. Despite extensive investigations, GBM′s resistance to the first‐line drug temozolomide (TMZ) remains a major challenge in clinical management. This study explores the molecular mechanisms underlying TMZ resistance in GBM, emphasizing the roles of DNA repair gene polymorphisms and intratumoral genetic heterogeneity.

**Methods:**

In this study, we collected 10 matched pairs of GBM surgical samples, including tumor tissues from the first and second surgeries, and proceeded with RNA‐Seq and Exome‐Seq. We performed pathway enrichment analysis and functional assays for key genetic variations in the DNA repair pathway to establish a mechanistic relationship between genetic changes and drug resistance. Sanger sequencing validated somatic variations before and after chemotherapy, and we analyzed changes in gene expression associated with DNA repair. The methylation status of the promoter region of the MGMT gene was analyzed, in addition to the effect of DNA repair genes on TMZ sensitivity in cells.

**Results:**

This study identified 20 novel somatic mutations that uniquely occurred in pre‐TMZ and post‐TMZ chemotherapy samples and were significantly related to DNA repair pathways (including base excision repair [BER] and nucleotide excision repair [NER]). Functional validation experiments confirmed that the alterations in the expressed variants had disrupted important repair mechanisms related to the survival of tumor cells. Notably, differential dysregulation of the NER and BER pathways (upregulated NER and inactivated BER) was observed in recurrent tumors, serving as a compensatory mechanism for TMZ resistance. Methylation of the MGMT gene promoter region has been linked to TMZ resistance, while intratumoral genetic heterogeneity might increase the chance of resistance. Importantly, our observations point toward an evolutionary event following TMZ treatment that incorporates selective pressures for repair‐deficient clones, resulting in a more aggressive fate for GBM. Cellular studies showed that the proliferation and migration ability of U87 cells were significantly elevated after the knockdown of XAB2, PNKP, and OGG1.

**Conclusion:**

This study represents the first comprehensive characterization of TMZ resistance in GBM based on integrated genetic, epigenetic, and functional validation approaches. In GBM, mechanisms of TMZ resistance are elucidated, with the interplay between the NER and BER pathways (compensatory regulation) being a key mechanism, alongside variations in DNA repair genes and intratumoral genetic heterogeneity. These findings highlight the importance of targeting the crosstalk between NER and BER pathways for GBM therapy, emphasizing the necessity of personalized treatment strategies and suggesting possible biomarkers for patient stratification by resistance profiles. Overall, these findings provide new avenues for developing personalized treatment strategies for GBM and can contribute to improving the prognosis of GBM patients.

## 1. Introduction

According to statistics from the American Brain Tumor Registry, glioblastoma (GBM, WHO Grade IV) is the most common type of primary brain tumor and is also one of the most malignant. The average survival time for GBM patients is reported to be only 9–12 months, with less than 2% surviving for more than 5 years [1]. The standard treatment regimen for GBM involves maximal surgical resection followed by a combination of temozolomide (TMZ) chemotherapy and radiotherapy. Clinical studies have reported that postoperative combined treatment can extend patient survival by an additional 4 months compared to radiotherapy alone [2]. However, a significant proportion of patients (~55%) are nonresponsive to TMZ chemotherapy in clinical settings.

GBM is one of the most lethal forms of cancer, with its invasive characteristics rendering surgical resection noncurative and making it one of the least responsive tumors to radiotherapy and chemotherapy. One major barrier to effective treatment is the tumor′s ability to rapidly acquire resistance to TMZ through mechanisms that remain incompletely understood, particularly how specific genetic changes interface with DNA repair pathways to drive this resistance. The current standard of care, comprehensive multimodal treatment, offers only moderate survival benefits to a subset of GBM patients. The development of effective treatments for GBM remains a significant challenge, which is attributed to the relatively low incidence of the tumor and its molecular heterogeneity [3, 4]. GBM is now one of the most molecularly characterized tumors. Genomic analyses have furthered our understanding of the complex molecular background of GBM and have accelerated the elucidation of its molecular heterogeneity, thus identifying potential rational targets for future targeted therapies [5]. Consequently, pharmacological inhibitors and modulators are currently under active investigation. Resistance to TMZ in GBM mainly relies on the tumor′s capacity to repair TMZ‐induced DNA damage before cell death starts. Building on this foundation, many laboratories are exploring additional chemotherapeutic measures, including [6] (1) antibody‐ and ligand‐targeted therapies against tumor cells that induce host antitumor responses or deliver toxic radionuclide conjugates, (2) gene therapies utilizing plasmids encoding toxic substances to selectively kill proliferating tumor cells, (3) antisense modulation and reverse mutation techniques to counter the expression of abnormal growth factors or inhibit their receptors, and (4) pharmacological strategies to disrupt abnormally activated signaling pathways in tumor cells, inhibit tumor angiogenesis, reduce invasiveness, and induce apoptosis. The exploration and refinement of these chemotherapeutic approaches will ultimately provide GBM patients with a highly personalized and safe treatment regimen, leading to long‐term control of the tumor.

TMZ primarily induces cytotoxic effects by alkylating guanine residues at the O6 position, resulting in DNA damage and hence tumor cell death; however, GBM cells use a range of DNA repair pathways: base excision repair (BER) and nucleotide excision repair (NER). More specifically, O6‐methylguanine‐DNA methyltransferase (MGMT) is a key repair enzyme that removes the toxic O6‐methylation that leads to TMZ resistance. Various studies have associated additional repair proteins, such as XAB2, PNKP, and OGG1, with modulating cellular responses to TMZ, albeit their precise roles in the evolution of GBM remain unknown. This study intends to enrich this understanding by utilizing high‐throughput sequencing of treatment‐naïve and treated GBM samples in the identification of repair gene variants contributing to resistance. Through integration of transcriptomic and exomic data, we will characterize the molecular evolution of TMZ resistance, revealing how DNA repair changes act to support tumor recurrence. Besides, we will address the influence of intratumoral genetic heterogeneity in shaping drug resistance, since several coexisting subclones may create niches that promote survival under chemotherapeutic stress. Understanding how these mechanisms act is crucial as they will inform further research endeavors toward the development of novel therapeutic strategies to circumvent TMZ resistance.

TMZ not only induces O6‐methylguanine (O6‐MeG) adducts but also generates abundant N7‐methylguanine and N3‐methyladenine lesions, which are primarily repaired by the BER pathway [7]; these lesions, though less cytotoxic, contribute significantly to genomic instability if unrepaired. Additionally, bulky and helix‐distorting DNA lesions, including those arising from oxidative stress or secondary damage, are substrates for NER‌ [8].

Notably, compensatory upregulation of NER in BER‐deficient tumors has been reported as a resistance mechanism in GBM and other cancers [9], highlighting the crosstalk between these pathways. This adaptive rewiring of DNA repair pathways may allow tumor cells to survive under chemotherapeutic stress, even when one pathway is compromised. Our findings of upregulated NER and inactivated BER in recurrent tumors align with this model, suggesting a compensatory mechanism contributing to TMZ resistance.

## 2. Materials and Methods

### 2.1. Sample Collection and High‐Throughput Sequencing Analysis

For 10 pairs of GBM tumors, tumor tissues were collected from initial and recurrent surgeries. Demographic characteristics, such as patient age, sex, tumor classification (IDH wild type/mutant and MGMT promoter methylation status), and other clinical characteristics, were recorded. Among these 10 paired samples, 6 pairs were fresh‐frozen surgical specimens, and the remaining 4 pairs were formalin‐fixed paraffin‐embedded (FFPE) specimens. Genomic DNA and total RNA were extracted under RNase‐free conditions to avoid contamination: All labware was treated with RNase inactivation solution (Thermo Fisher Scientific), RNA extraction was performed using the RNeasy Mini Kit (Qiagen) supplemented with RNase inhibitor (40 U/*μ*L), and all steps were conducted in a dedicated RNA workspace to minimize RNase exposure. Genomic DNA was extracted using the DNeasy Blood & Tissue Kit (Qiagen), while DNA from peripheral blood was used as a control. In addition, the pathological sections for the different tumor regions (three to five regions per tumor) were analyzed to assess intratumoral genetic heterogeneity. For all 10 pairs of GBM samples, transcriptome sequencing (RNA‐Seq, PE150bp, Illumina NovaSeq 6000) was performed. Of the 10 pairs, 6 fresh‐frozen sample pairs underwent whole‐exome sequencing (Exome‐Seq, target depth of 150–200×), which yielded high‐quality, high‐coverage sequencing data for core bioinformatics analysis and result presentation. The other four FFPE sample pairs underwent Exome‐Seq with a target depth of 100–150×; however, these samples showed severe DNA degradation, low library complexity, and poor sequencing data uniformity, which may lead to erroneous or misleading results. Therefore, the sequencing data of these four FFPE samples were not included in the core results analysis, while their Sanger sequencing validation data for somatic variants and MGMT promoter methylation detection results were still incorporated into the relevant supplementary analyses. Somatic variants identified by Exome‐Seq were filtered using standard bioinformatics pipelines, including alignment with BWA‐MEM, variant calling with GATK, and annotation with ANNOVAR. For comparison, selected variants were validated by PCR amplification and Sanger sequencing of peripheral blood gDNA. A comparative method confirmed variants that were present in pre‐TMZ or post‐TMZ treatment samples only.

Of the 10 pairs, 6 underwent whole‐exome sequencing (Exome‐Seq, target depth of 150–200×), while the other 4 underwent Exome‐Seq with a target depth of 100–150×. The lower sequencing depth in the latter group was due to the use of FFPE tissue samples, which typically yield lower quality DNA and reduced library complexity compared to fresh‐frozen tissues. Despite the lower depth, 100× coverage is considered sufficient for reliable somatic variant detection in cancer studies, particularly when combined with targeted validation methods. To enhance sensitivity in these samples, peptide nucleic acid (PNA)–PCR was employed to selectively amplify variant alleles and suppress wild‐type background, enabling detection of somatic variants at frequencies as low as ~10%.

Germline variant calling used peripheral blood DNA as a control. However, to mitigate biases from clonal hematopoiesis (CHIP), variants in established CHIP‐associated genes (e.g., DNMT3A, TET2, and ASXL1) were carefully evaluated. Only variants with allele frequencies close to 50% were considered germline; those with subclonal frequencies (typically < 30%) and occurring in older individuals were flagged as potential CHIP‐related and excluded from germline interpretation [10].

### 2.2. Clinical Data Repository Construction

A clinical database was created to store data about the patients: their ages, sex, tumor location, molecular classifications, methylation status, IDH mutation status, chromosomal abnormalities, dates of initial and recurrent surgery, and the survival outcomes. The data were next employed to correlate the variations in genetic features with clinical characteristics.

### 2.3. Ethical Approval

The study was conducted in accordance with the ethical principles of the Declaration of Helsinki. Ethical approval was obtained from the Institutional Review Board of Dongguan People′s Hospital (Approval Number: AF‐97‐04). Prior to sample collection, written informed consent was obtained from each participant or their legal representative.

### 2.4. Variant Signature Profile Analysis

TMZ′s mutational signature is characterized by C>T/G>A base transitions in CpC and/or CpT islands. To assess these mutational events, Exome‐Seq data were used to analyze the variant frequencies in samples obtained before and after treatment with TMZ.

### 2.5. Gene Set Enrichment Analysis (GSEA)

DNA repair‐associated proteins were divided into categories based on their action in BER, NER, MMR, and HR. Pathway enrichment comparisons for pre‐ and post‐TMZ treatment samples derived from RNA‐Seq were computed using GSEA (V5.0, Broad Institute). Genes were clamped by fold change in expression, and significantly upregulated repair pathways were identified by FDR‐corrected *p* values (*q* < 0.05).

### 2.6. Intratumoral Genetic Heterogeneity Identification

After treatment with TMZ, there have been reports of high‐frequency somatic mutations in Samples M1 and 3563. For Sample 3563, the elevated mutational burden is consistent with the correlation between TMZ‐induced genomic instability and tumor aggressivity, as evidenced by the functional validation of DNA repair gene variants (e.g., PNKP and OGG1) in promoting invasive phenotypes. To examine spatial heterogeneity among three distinct tumor regions per sample, we performed Exome‐Seq using FFPE‐derived material. Owing to an average Exome‐Seq depth of 191×, PNA‐PCR was utilized as an additional measure to enhance sensitivity by selectively amplifying variant alleles and blocking the wild‐type alleles, thus allowing the detection of somatic variants at a frequency of as low as ~10%. Further validation of the somatic mutations was achieved by Sanger sequencing at frequencies of 0.5% or less. For the 20 variants post‐TMZ treatment, PNA‐PCR was used to analyze their existence in pre‐TMZ treatment specimens, ensuring that mutations were acquired instead of missed at a lower frequency during the sequencing process. The pathogenicity of identified somatic variants was assessed using HDivPred, a tool specifically optimized for predicting the functional impact of variants in DNA repair genes with high sensitivity and specificity for tumor‐related mutations [11]. This tool was selected due to its validated performance in GBM and chemoresistance‐associated variant analysis, effectively distinguishing pathogenic mutations from benign polymorphisms. The driver mutations were then compared against the COSMIC database V76 for tumor evolutionary dynamics, and we constructed phylogenetic trees representing the clonal relationships in GBM evolution.

### 2.7. Cell Culture and Functional Validation

U87, A172 GBM cell lines, NHA, and primary patient‐derived GBM cells were cultured in DMEM with 10% fetal bovine serum (FBS) and 1% penicillin–streptomycin at 37°C with 5% CO_2_.

Cells were treated with MGMT inhibitor O‐6‐benzylguanine (10 *μ*M) for 2 h before being treated with TMZ (20–120 *μ*M).

Cell viability was measured daily using MTT, and apoptosis was assessed with Annexin V/PI staining and flow cytometry. The effects on proliferation and migration due to the knockdown of XAB2, PNKP, and OGG1 were assessed by scratch wound‐healing and Transwell invasion assays.

## 3. Results

### 3.1. Identification of Genes Related to DNA Repair Pathways

**Figure 1:**
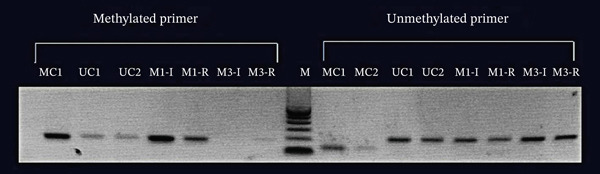
Comparison of MGMT promoter region methylation status between Patients M1 and K3. MC represents the methylated sample control, whereas UC represents the unmethylated sample control. M1‐I: M1‐Initial, M1‐R: M1‐Recurrence, M: marker.

Using the Ion Proton platform by Life Technologies, exome sequencing and subsequent bioinformatics analysis were performed on six paired samples of cancerous tissues. The sequencing libraries were prepared using PE100bp. An average sequencing depth of 191.1× was obtained with a whole‐exome off‐target read rate of less than 10% and sequencing uniformity above 85% (Table 1). We also analyzed DNA repair pathway‐related genes and found 20 private somatic variants present only in the pre‐TMZ or post‐TMZ treatment samples, all validated using Sanger sequencing. To assess the novelty of recurrent mutations, variants were cross‐referenced with COSMIC (V101), gnomAD, and ClinVar. Several recurrent mutations in BER and NER pathway genes (e.g., ∗XAB2∗, ∗PNKP∗, and∗OGG1∗) were absent from gnomAD and ClinVar, and underrepresented or absent in COSMIC, suggesting they are novel or extremely rare in existing cancer cohorts‌. This is consistent with findings that mutation recurrence correlates with oncogenic activity and poor prognosis [12]. Given that existing databases often contain biases from targeted panels and duplicate entries, our use of whole‐exome sequencing from matched pre‐ and posttreatment samples provides a more accurate assessment of true recurrence [12]. Among these, recurrent mutations appeared in BER genes (PNKP and OGG1) and NER genes (XAB2 and ERCC1/2) (Table 2), which need further validation in a larger GBM sample set. Comparison of pre‐TMZ and post‐TMZ treatment samples revealed the most significant upregulation of mRNA levels in the NER pathway genes ERCC1/2 and XAB2 (fold change: 2.5–3.1; *p* < 0.01), followed by the BER pathway genes PNKP and OGG1 (fold change: 1.8–2.3; *p* < 0.05) (Figure 1). This upregulation reflects a relative increase in recurrent tumors compared to initial tumors, likely representing an adaptive transcriptional response in surviving subclones under TMZ pressure. However, this does not contradict the observation of lower baseline expression of PNKP and OGG1 in GBM cell lines compared to normal glial cells (HEB), which reflects inherent deficiencies in DNA repair capacity in tumor cells. To support this finding, 607 GBM samples were examined from the TCGA database. To assess the broader relevance of the observed expression changes, we analyzed 607 GBM samples from the TCGA database. These samples revealed a low frequency of SNVs (< 3%) in XAB2, PNKP, and OGG1, suggesting that the expression changes observed in our cohort may arise due to TMZ exposure and adaptive responses, rather than pre‐existing mutations [13]. These samples revealed a low frequency of SNVs (< 3%) in these genes, which implies that their expression changes might arise due to exposure to TMZ instead of pre‐existing mutations.

**Table 1:** Exome sequencing quality of six pairs of tumor samples.

Sample name	Mapped reads	On target	Mean depth	Uniformity
3563‐I	54,909,159	92.56%	137.9	87.70%
3563‐R	75,413,917	94.55%	200	89.14%
K3‐I	83,584,409	94.41%	200	87.21%
K3‐R	76,122,334	93.90%	222.4	86.82%
M1‐I	79,535,718	93.55%	187.4	88.52%
M1‐R	88,547,963	92.74%	215.8	87.41%
M3‐I	75,768,125	92.40%	192	91.35%
M3‐R	81,560,063	94.48%	188.1	87.33%
KVS1‐I	77,829,026	93.26%	173.6	89.54%
KVS1‐R	66,369,092	94.91%	182.8	85.62%
KVS4‐I	76,798,575	92.11%	196.3	87.03%
KVS4‐R	81,980,940	94.26%	197	86.19%

**Table 2:** DNA repair‐associated genes with coding region variants.

MMR		DSBR		NER		BER	
Gene	Patient	Gene	Patient	Gene	Patient	Gene	Patient
MLH1	3563‐R M1‐R	RAD50	K3‐R	ERCC1	3563‐R	PNKP	3563‐R
MSH2	3563‐R M1‐R M3‐R	LIG4	3563‐R	ERCC2	K3‐R KVS1‐R	LIG1	3563‐R
MSH3	3563‐R			XAB2	3563‐R	LIG3	K3‐R
MSH6	3563‐R M1‐R KVS4‐R			GTF2H4	K3‐R KVS1‐R KVS4‐R	OGG1	3563‐R K3‐R KVS4‐R
PMS1	3563‐R			DDB1	3563‐R	PARP1	3563‐R
MLH3	M1‐R 3563‐R			ERCC6	3563‐R	PARP3	M1‐R K3‐R M3‐R

### 3.2. Methylation Status of the MGMT Gene Promoter Region

**Figure 2:**
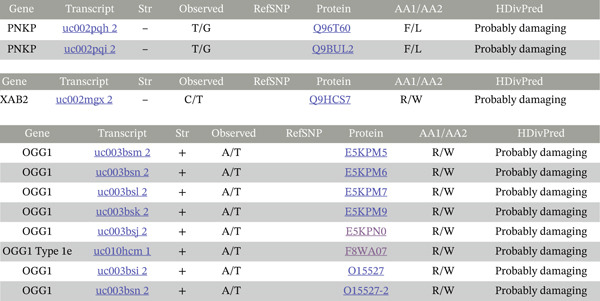
Polyphen‐2 and SIFT predictions of potential functional mutations. Polyphen‐2 predictions indicate that the coding mutation is “probably damaging.”

**Figure 3:**
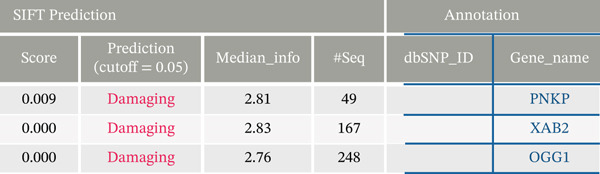
SIFT predictions consistently show the coding mutations as “damaging.”

Methylation of the MGMT gene promoter region has recently been recognized to contribute to an extensive increase in variants seen after TMZ chemotherapy. Among the gathered samples, M1′s recurrent tumor was characterized by a high‐frequency variant state with somatic mutations increasing by 3.2‐fold from pre‐TMZ to post‐TMZ treatment (Figure 2). This marked increase in mutational burden aligns with our observation that TMZ‐induced repair‐deficient clones contribute to a more aggressive GBM phenotype, as supported by our cellular data showing enhanced migration and invasion upon knockdown of key DNA repair genes (XAB2, PNKP, and OGG1). On the other hand, M3′s recurrent tumor did not exhibit any significant changes in mutation frequency. We used methylation‐specific PCR (MSP) to examine 12 tumor DNA samples, with M1‐R showing hypermethylation of the MGMT promoter region, while M3‐R was unmethylated (Figure 3). The findings reveal that the MGMT methylation status correlates with mutational burden and clonal evolution after TMZ treatment (Figure 1).

### 3.3. Functional Validation of DNA Repair Gene Variants

**Figure 4:**
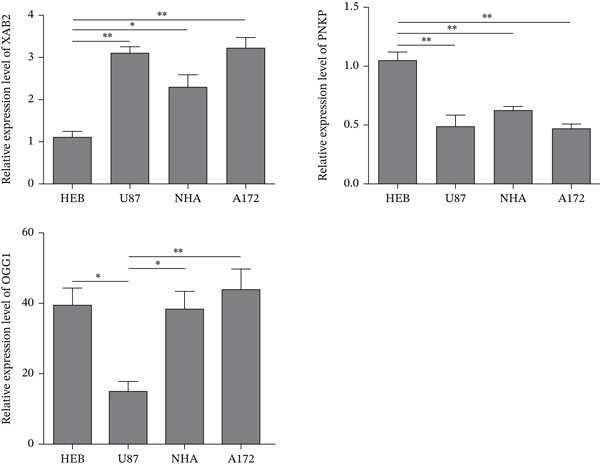
Cultivate HEB normal human glial cells and three GBM cell lines, U87, NHA, and A172. First, add the MGMT inhibitor O‐6‐benzylguanine to coculture with the cells, and then treat with TMZ at different concentration gradients (20–120 *μ*M) to detect the expression levels of XAB2, PNKP, and OGG1. The horizontal lines with asterisks indicate that there is a significant difference between the two sets of data at both ends of the lines.

In addition, we evaluated the expression of XAB2, PNKP, and OGG1 proteins in U87, NHA, and A172 GBM cell lines, as well as in HEB cells, which are considered normal gliocytes. Cells were preincubated for 2 h with the MGMT inhibitor O‐6‐benzylguanine (10 *μ*M), followed by 20–120 *μ*M of TMZ. XAB2 mRNA expression was then significantly elevated in U87, NHA, and A172 when compared with HEB cells (*p* < 0.05, *p* < 0.01) (Figure 4), consistent with the upregulation of its protein level (Figure 1). PNKP expression levels in U87, NHA, and A172 were significantly lower than in HEB cells. This reflects the baseline deficiency in BER capacity commonly observed in GBM, which may be partially compensated by transient upregulation in surviving clones after TMZ exposure. Therefore, inhibition of PNKP expression may enhance the DNA repair capabilities for single‐strand breaks. To confirm, western blot analysis was performed to show whether a loss of these proteins contributes to TMZ resistance (*n* = 3 replicates). The polyclonal antibody readily detected the PNKP and XAB2 proteins in HEB cells and all three GBM cell lines with slight differences. U87, NHA, and A172 cells expressed higher levels of XAB2, with relative fluorescence intensities 3.5‐, 4.2‐, and 5.3‐fold higher than HEB cells, respectively. In contrast, PNKP levels were significantly lower in U87, NHA, and A172 cells than in HEB cells (*p* < 0.01) (Figure 4). Gene knockdown efficiency was verified by RT‐PCR, and western blot analysis further confirmed that TMZ treatment modulated the expression levels of DNA repair proteins (XAB2, PNKP, and OGG1) in the tested cell lines.

### 3.4. Proliferation and Migration Assays Following Knockdown of DNA Repair Genes

**Figure 5:**
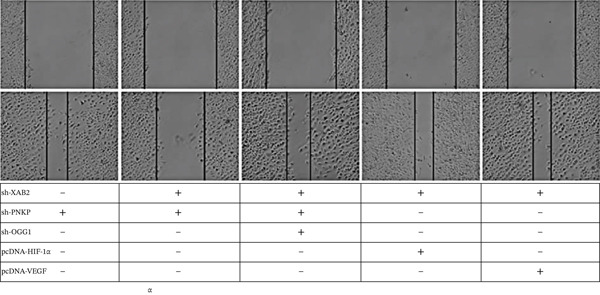
Cell scratch assay. Migration ability of U87 after knocking down XAB2, PNKP, and OGG1. Significantly enhances U87 cell migration.

Cell migration assays: The knockdown of XAB2, PNKP, and OGG1 markedly promoted migration of GBM U87 cells through a 40.5*%* ± 4.2*%* reduction in scratch wound closure time (*p* < 0.001) (Figure 5).

In vivo Transwell invasion assays: The knockdown of XAB2, PNKP, and OGG1 in cells has been shown to increase the invasion rate by an impressive 2.1‐fold (*p* < 0.01), which indicates a more aggressive phenotype.

MTT cell viability assays: Following XAB2 knockdown, cells that were then exposed to TMZ showed significantly greater survival, increasing from a 2.5‐fold baseline (*p* < 0.001), indicating that loss of XAB2 enhances resistance to TMZ‐related cell toxicity.

## 4. Discussion

TMZ remains the primary chemotherapy agent for GBM. Unfortunately, however, this agent often falls short in effectiveness owing to the much‐feared chemoresistance, which greatly limits patient outcomes. One of the principal ways in which TMZ resistance occurs is through several DNA repair pathways that help shield against TMZ‐induced alkylation damage. The MGMT enzyme is a very potent DNA repair enzyme that confers resistance to TMZ by transferring O6‐MeG adducts to its cysteine residues, thus preventing apoptosis while propelling tumor cell survival [14]. The degree of TMZ effectiveness is largely determined by the methylation of the MGMT promoter. This methylation curbs MGMT transcription, thus allowing the tumor to be more sensitive to alkylating agents [15]. In our study, however, we demonstrate that TMZ resistance is not solely due to MGMT activity; other DNA repair pathways, including BER and NER, also allow tumor cells to survive under the stress imposed by TMZ. The key somatic mutations in DNA repair genes identified by our study included but were by no means limited to XAB2, PNKP, and OGG1. XAB2, a central player in the NER pathway, showed a significant upregulation in recurrent GBM samples, implying that the NER activity was enhanced and compensated for TMZ‐induced damage to GBM cells, allowing them to evade apoptosis [16]. From our findings, we conclude that the alterations to the NER and BER pathways significantly contribute to the resistance of tumors to TMZ treatment. In recurrent tumors treated with TMZ, NER‐associated pathways involving ERCC1, ERCC2, and XAB2 were found to be upregulated, which implies a better ability to repair bulky DNA adducts. On the other hand, the BER pathway genes were mutated and thereby inactive, leading to oxidative DNA damage; molecules involved include PNKP and OGG1, which promote tumor aggressiveness. Our hypothesis is that this differential regulation of the NER and BER pathways may serve as compensatory mechanisms allowing tumor cells to survive the chemotherapeutic attack. This observation is consistent with past reports describing how BER‐deficient tumors depend on alternative pathways to repair DNA and survive chemotherapy‐induced cell death. It is important to note that while XAB2 is upregulated in recurrent tumors—consistent with enhanced repair capacity—its knockdown paradoxically increased TMZ resistance. This may reflect a dual role of XAB2 beyond direct repair. Recent studies show that XAB2 is released from R‐loops during DNA repair and becomes more mobile after damage induction, suggesting a regulatory role in transcription‐coupled repair rather than a structural one [17]. Its loss may lead to prolonged RNA Polymerase II stalling and R‐loop accumulation, promoting genomic instability and clonal evolution under chemotherapeutic stress. Furthermore, XAB2 interacts with key TC‐NER factors (CSA, CSB, and XPG)‌ and may influence splicing and transcription fidelity [18]. Thus, while its upregulation likely reflects an adaptive response, its knockdown may promote a more aggressive, therapy‐resistant phenotype through noncanonical mechanisms. In contrast, BER‐associated proteins were found to be highly dysregulated in PNKP and lowly dysregulated in OGG1 in the recurrent GBM cells [19]. It is possible that BER‐deficient tumors rely on alternative NER pathways, thus facilitating tumor evolution under chemotherapeutic pressure [20]. It is noteworthy that although PNKP expression is lower in GBM cell lines than in normal glial cells, its knockdown further enhances migration and invasion. This may reflect a threshold effect, where further reduction in an already compromised repair system leads to excessive DNA damage and activation of proinvasive signaling. PNKP plays a critical role in processing DNA ends during repair and in Okazaki fragment maturation [21]. Loss of PNKP function can result in persistent single‐stranded DNA gaps during S phase, which are known to trigger replication stress and genomic instability [22]. These conditions may promote epithelial–mesenchymal transition (EMT)–like changes and enhance metastatic potential, rather than induce cell death. This is consistent with the concept that moderate DNA damage promotes tumor evolution, while only severe damage leads to apoptosis. These findings emphasize the complex nature of TMZ resistance mechanisms and highlight the necessity for therapeutic strategies directed not only toward MGMT but also toward other repair pathways. RNA‐Seq analysis of paired samples obtained before and following treatment further confirmed that upregulation of the genes in NER (ERCC1, ERCC2, and XAB2) in recurrent tumors was evident. At the same time, downregulation of BER was caused by reduced expression (downregulation) of PNKP and OGG1. The ERCC1/2 proteins remove bulky DNA adducts, and their upregulated expression in TMZ‐resistant specimens is congruent with the data that indicate NER was activated to repair TMZ‐induced lesions during cellular recovery from treatment [23]. Loss of both PNKP and OGG1 expression is consistent with prior studies indicating that tumors compromised for BER develop resistance through the use of alternative repair mechanisms [24]. Thus, we describe a novel insight into GBM chemotherapy resistance that is associated with the differential regulation of the NER and BER pathways, thereby corroborating the assumption that TMZ treatment exerts a strong selective pressure on GBM cells that promotes the emergence of resistant subclones with enhanced repair capacity [25]. Further complicating the picture of TMZ resistance is the emergence of heterogeneity within the tumor. The selective pressures of chemotherapy create a unique situation in which highly proliferative, treatment‐resistant clones evolve, leading to tumor relapses [26]. In line with this study′s observations, post‐TMZ‐treated tumors from M1 and 3563 had higher mutation burdens, prevalently manifesting as C>T/G>A transitions in CpC and CpT island regions, a hallmark of the mutational signature described as TMZ‐induced genomic instability [27]. This increase in somatic mutations suggests that TMZ not only confers a selective advantage to the resistant clones but also accelerates the genetic evolution of the tumor toward more aggressive phenotypes [28]. The interaction between DNA repair defects and clonal selection further highlights the need for precision medicine integration into therapeutic decision‐making to account for tumor heterogeneity. Despite the significance of our findings, we acknowledge that the small sample size (*n* = 10) limits the statistical power and generalizability of our conclusions. Additionally, the rarity of paired, well‐characterized GBM specimens limits the generalizability of our findings, and future studies will expand the cohort size to validate these results against a larger dataset. It is important to note that blood‐derived DNA may harbor somatic mutations due to clonal hematopoiesis, particularly in aging individuals. These variants can mimic germline polymorphisms and lead to misclassification if not properly addressed [10]. The study should be interpreted as exploratory, with the primary goal of identifying candidate genes and pathways involved in TMZ resistance. Expanding the cohort with a larger number of paired pre‐ and posttreatment GBM samples would offer a clearer understanding of the mutational landscape underlying resistance to TMZ. While our study identified critical somatic mutations in DNA repair genes, more functional validation using isogenic GBM models with engineered knockouts of XAB2, PNKP, and OGG1 is required to properly ascertain their roles in chemoresistance. While our study identified critical somatic mutations in DNA repair genes, more functional validation using isogenic GBM models with engineered knockouts of XAB2, PNKP, and OGG1 is required to properly ascertain their roles in chemoresistance [29]. Using peripheral blood DNA as a control offers practical advantages, including easy accessibility, minimal invasiveness, and its utility as a germline reference to distinguish somatic mutations from germline variants. However, it has inherent limitations: (1) Germline variants may interfere with somatic mutation calling, which we mitigated by filtering variants with allele frequencies close to 50% (as described in the Methods section); (2) post‐TMZ treatment blood may have insufficient circulating tumor DNA (ctDNA) to reflect tumor‐specific changes, limiting its ability to capture dynamic genomic alterations in recurrent tumors. In addition, integrating proteomics and metabolomics with transcriptomics may provide deeper insight into the alterations in repair pathways and their far‐reaching ramifications on tumor biology [19]. Targeting the DNA repair vulnerabilities of TMZ‐resistant GBM may offer therapeutic opportunities. Combination therapies that target compensatory DNA repair pathways by simultaneously inhibiting both NER and BER are therefore expected to enhance the efficacy of TMZ by minimizing the probability of developing acquired resistance [20]. Furthermore, personalized treatment strategies based on tumor‐specific repair gene mutations and MGMT methylation status may help optimize treatment selection and improve patient prognosis [30]. Future studies should also delineate pharmacological inhibitors targeting DNA repair factors such as XAB2, ERCC1, or other key targets to counteract TMZ resistance mechanisms [22]. In conclusion, the present study comprehensively elucidates the crosstalk between DNA repair pathways and TMZ resistance in GBM. We show that treatment failure and tumor recurrence are associated with active NER‐mediated repair mechanisms coupled with downstream inhibition of BER and that MGMT methylation is a key but insufficient determinant of treatment response. Such findings reveal an urgent need for combined therapeutic strategies targeting diverse mechanisms of chemoresistance in GBM to improve treatment outcomes [23].

## Author Contributions

We declare that all the listed authors have participated actively in the study and all meet the requirements of authorship. Dr. W.C. designed the study and wrote the paper. Dr. C.L. undertook the data acquisition and analysis. Dr. Y.L. managed the literature searches and clinical studies. Dr. H.H. contributed to the correspondence and paper preparation. All authors reviewed the manuscript.

## Funding

This work was supported by the Guangdong Provincial Medical Science and Technology Research Fund (Grant No. B2023157).

## Ethics Statement

The study protocol was reviewed and approved by the Institutional Review Board of Dongguan People′s Hospital (Approval Number: AF‐97‐04). Prior to enrollment, written informed consent was obtained from all participants or their legal representatives.

## Consent

The authors have nothing to report.

## Conflicts of Interest

The authors declare no conflicts of interest.

## Data Availability

The datasets used or analyzed during the current study are available from the corresponding author on reasonable request.
